# Randomized controlled trial of a brief dyadic cognitive-behavioral intervention designed to prevent PTSD

**DOI:** 10.3402/ejpt.v4i0.21572

**Published:** 2013-08-26

**Authors:** Alain Brunet, Isabeau Bousquet Des Groseilliers, Matthew J. Cordova, Josef I. Ruzek

**Affiliations:** 1 Psychosocial Research Division, Douglas Institute Research Center, Verdun, Québec, Canada; 2 Department of Psychiatry, McGill University, Montréal, Québec, Canada; 3 Université du Québec à Montréal, Montréal, Québec, Canada; 4 Douglas Institute Research Center, Verdun, Québec, Canada; 5 Pacific Graduate School of Psychology, VA Northern California Health Care System, Martinez Outpatient Clinic—Mental Health, Martinez, CA, USA; 6 Dissemination and Training Division, National Center for PTSD, VA Palo Alto Health Care System, Menlo Park, CA, USA

**Keywords:** Traumatic stress, secondary prevention, motor vehicle accidents, cognitive-behavioral (CBT) interventions, Canadians

## Abstract

**Background:**

There is a dearth of effective interventions to prevent the development of post-traumatic stress disorder (PTSD).

**Method:**

We evaluated the efficacy of a brief dyadic two-session cognitive-behavioral intervention through a controlled trial involving trauma-exposed individuals recruited at the hospital's emergency room. Participants were randomly assigned to either the dyadic intervention group (*n=*37) or to a waiting list (assessment only) group (*n=*37).

**Results:**

In an intent-to-treat analysis, a time-by-group interaction was found, whereby the treated participants had less PTSD symptoms at the post-treatment but not at the pre-treatment compared to controls. Controlling for the improvement observed in the control participants, the intervention yielded a net effect size of *d=*0.39.

**Conclusions:**

A brief, early, and effective intervention *can* be provided by nurses or social workers in hospital settings, at a fairly low cost to individuals presenting to the emergency room as the result of trauma exposure.

In most societies, exposure to traumatic events is ubiquitous and associated with significant levels of post-event problems, including post-traumatic stress disorder (PTSD) (Collins et al., 2011). Despite the fact that trauma exposure and PTSD represent an important public health problem, attempts at secondary prevention are few and have generated more disappointment than hope (Kearns, Ressler, Zatzick, & Rothbaum, 2012). The most widely available early intervention, single-session individual stress debriefing, has been found to be ineffective in preventing PTSD and depression (Bisson, McFarlane, Rose, Ruzek, & Watson, 2009). Psychological first aid has been manualized but not yet evaluated (Vernberg et al., 2008). Translational approaches are promising (e.g., Poundja, Sanche, Tremblay, & Brunet, 2012) but still in their infancy.

Currently, the best validated early intervention is an early and intensive trauma-focused cognitive-behavioral approach (Kornor et al., 2008; Rothbaum et al., 2012). A strength of this intervention is the application of well-validated exposure therapy methods in the context of early preventive intervention. However, this may also represent a limitation, in that there is a risk that exposure may be applied inappropriately by non-specialist providers if significant levels of training and support are not provided.

There are, in fact, many other possible approaches to cognitive-behavioral early intervention that require scientific exploration (Ruzek, 2006), especially those that target social support processes (Olff, 2012). Perceived lack of social support is a risk factor for development and persistence of PTSD (Brewin, Andrews, & Valentine, 2000). Negative social support reactions from others have been found to predict PTSD symptomatology (Bolton, Glenn, Orsillo, Roemer, & Litz, 2003) and deterioration of social support has been associated with declining mental health in disaster survivors.

Cordova, Ruzek, Benoît, and Brunet (2003) described a brief dyadic cognitive-behavioral intervention designed to target social support processes following trauma exposure. This intervention aims at improving the patient and significant other's knowledge of the process of adjustment to stressful experiences; to increase tolerance for negative emotions; to increase rates of initiation of talking about the trauma and its effects, and to decrease social constraint behaviors in response to disclosure of trauma-related material.

## Objectives and hypotheses

The primary objective of this study was to empirically test the efficacy of a brief dyadic secondary prevention intervention, as described by Cordova et al. (2003), in reducing PTSD symptomatology in the weeks and months following trauma exposure. We hypothesized that there would be a time-by-group interaction whereby self-reported PTSD symptoms would be less severe in the intervention group than in the no-intervention control group 3 months after trauma exposure, but not before or during treatment. A secondary objective was to examine whether this intervention can help decrease perceived negative social support in a dyad. Thus, we hypothesized that there will be a significant decrease in the amount of participants perceiving negative social support in the intervention group but not in the control group. Finally, the efficacy of the intervention will be tested relative to other outcome measures, such as post-treatment PTSD diagnosis, psychiatric comorbidity, substance use, and occupational functioning.

## Methodology

### Study design and participants

Participants were recruited over 24 months at the emergency rooms of two Montréal public hospitals. Using a modified version of the randomly permuted blocks method (Fleiss, 1986), participants in this randomized controlled trial with three measurement times (pre-treatment, mid-treatment, and post-treatment) were blindly assigned with a 50% chance of being assigned to one of two treatment conditions: intervention or no-intervention with periodic assessments.

#### Inclusion/exclusion criteria

Participants had to have experienced in the last 10 days a life-threatening event that elicited a peritraumatic reaction of fear, helplessness, or horror. This corresponds to the A1 and A2 criteria. Participants were excluded if they did not speak either French or English; had or were suspected of having a traumatic brain injury; had a lifetime diagnosis of psychosis, substance or alcohol dependence, bipolar disorder, or mental retardation; had been clinically depressed in the last 2 years; were taking psychotropic medication at the onset of the study; were injured to the extent that they could not participate in the study; lived outside the Montreal metropolitan area; did not have a significant other (a friend, a spouse or another family member) to bring to the therapy session, or did not succeed in making an appointment with the therapist within 30 days after trauma exposure.

#### Drop-outs

**Fig. 1:**
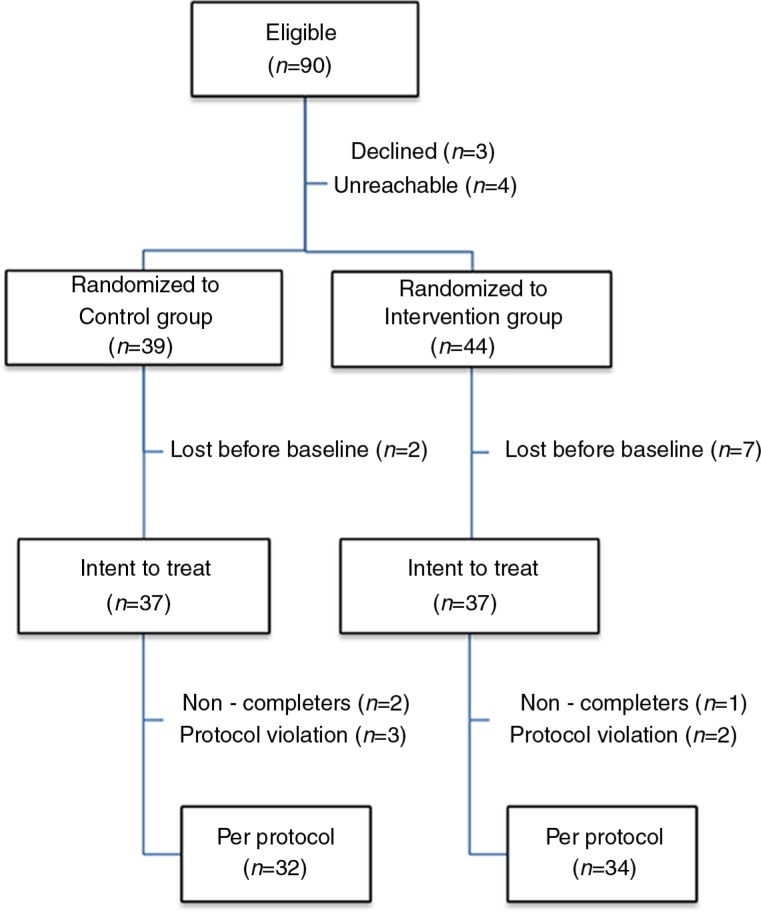
Flow chart of the study participants. Note. Several randomized participants were lost before baseline, most of them did not return their baseline symptom assessment and/or failed to find a time to meet the therapist in a timely manner. The intent to treat sample completed at least two symptom assessments and received at least one intervention session. Protocol violation in the control group entailed the following exclusion criteria : initiating trauma-related court litigation in one case and a loss of consciousness undiagnosed at the time of recruitment in two cases. Protocol violation in the intervention group entailed coming alone to the dyadic intervention and being diagnosed with a terminal illness during the study.

As shown in Fig. 1, 90 participants were deemed eligible. A total of 83 were randomized. Ten participants in the intervention group, and seven in the control group, left the study before the end, yielding a drop-out rate of 20%. The main reason for dropping out was the tight schedule for conducting the intervention (i.e., in the third week after trauma exposure). This often led to scheduling problems between the therapist (available only on a part-time basis), the study participants and their intervention partner. Six participants were excluded during the analyses because they did not follow the instructions properly or because of too much missing data.

#### Socio-demographic data

As shown in Table 1, the intent-to-treat (ITT) sample was composed of 74 mostly Caucasian participants, with an average age of 36.33 years (range: 19–63, *SD:* 10.82) and a median/modal annual family income of 30,000–50,000 CAD$. Participants had a median 13 years of schooling and 75% of them were in a relationship. They had been admitted to the hospital's emergency room as a result of one of the following events: motor vehicle accident (*n=*41), work accident (*n=*12), leisure accident (*n=*10), or physical assault (*n=*11). There were no between-group differences on the type of traumatic events reported (not shown in Table 1), socio-demographic variables, injury severity, trauma measures, or amount of negative social support at the pre-treatment assessment.

**Table 1:** Socio-demographic data: intent-to-treat population

	Group				
	
	Treated (*n*=37) *M* (SD)	Untreated (*n*=37) *M* (SD)	*df*	*t*	*p*
Age	37.97 (12.58)	34.68 (9.16)	72	1.29	0.20
Peritraumatic distress^1^	22.70 (8.08)	23.11 (7.40)	72	0.23	0.82
Peritraumatic dissociation^2^	23.19 (6.87)	21.92 (7.59)	72	−0.75	0.45
Injury severity^3^	7.62 (6.74)	6.54 (5.00)	72	−0.78	0.44
Negative support^4^	23.35 (8.27)	24.24 (8.95)	71	0.44	0.66
	*n* (%)	*n* (%)	*df*	χ^2^	*p*
Annual household income					0.63^6^
Up to $15,000 CAD	3 (9%)	7 (20%)			
$15,001–30,000	8 (22%)	7 (20%)			
$30,001–50,000	12 (33%)	11 (31%)			
$50,001–70,000	5 (14%)	6 (17%)			
$70,001–90,000	4 (11%)	3 (9%)			
$90,001 and above	4 (11%)	1 (3%)			
Undisclosed^5^	1	2			
Gender			1	1.96	0.16
Female	20 (54%)	14 (38%)			
Male	17 (46%)	23 (62%)			
Ethnicity			1	0.11	0.74
Caucasian	32 (86%)	31 (84%)			
Other	5 (14%)	6 (16%)			
Marital status			1	1.26	0.26
Married/living together	25 (69%)	26 (81%)			
Other	11 (31%)	6 (19%)			
undisclosed^5^	1	5			
Education level			1	2.26	0.13
College graduate or less	24 (65%)	29 (81%)			
Some university and more	13 (35%)	7 (19%)			
Undisclosed^5^	N/A	1			

1 Peritraumatic Distress Inventory;

2 Peritraumatic Dissociative Experience Questionnaire;

3 Injury Severity Score;

4 Social Constraints Scale;

5 excluded from the statistical test;

6 Fisher's exact test.

Some measures could only be computed in the per-protocol sample due to the fact that the data were collected at the last visit, and therefore not collected for the ITT sample. This was the case for psychiatric comorbidity. The two study groups included a similar number of individuals with prior psychopathology (15 vs. 12), χ^2^(1, 66)=0.30, *p=*0.38. Furthermore, the intervention and control groups (respectively) did not differ on current or past psychiatric comorbidity at the pre-test: major depressive episode (11 vs. 10), manic episode (1 vs. 1), panic disorder with (1 vs. 0) and without agoraphobia (1 vs. 3), agoraphobia without panic disorder (1 vs. 1), social phobia (1 vs. 2), obsessive–compulsive disorder (2 vs. 2), generalized anxiety disorder (0 vs. 3), anorexia nervosa (0 vs. 1), or bulimia (0 vs. 3).

### Study measures

#### Trauma measures

The Peritraumatic Distress Inventory (PDI) (Brunet et al., 2001; Jehel, Brunet, Paterniti, & Guelfi, 2005) is a 13-item self-report measure with items ranging from 0 (not at all true) to 4 (extremely true). A score of 3 or 4 on item 1 (fear), 4 (helplessness), or 10 (horror) was used as the threshold to ascertain trauma exposure according to the DSM-IV-TR criteria. The Peritraumatic Dissociative Experience Questionnaire (PDEQ) (Birmes et al., 2005; Marmar, Weiss, & Metzler, 1997), a 10-item self-report measure with items ranging from 1 (not at all true) to 5 (extremely true), was used to further gauge the severity of the trauma response across the study groups. The Impact of Event Scale-Revised (IES-R) (Brunet, St-Hilaire, Jehel, & King, 2003; Weiss & Marmar, 1997) is a dimensional 22-item self-report measure with items ranging from 0 (not at all true) to 4 (extremely true) assessing the intensity of PTSD symptoms. The IES-R was used as the main outcome because it allows for a quick and valid measurement of the intensity of trauma symptoms in the last 7 days. The Clinician-Administered PTSD Scale (CAPS) (Blake et al., 1995), a semi-structured interview assessing the frequency and intensity of the 17 symptoms of PTSD, was used by trained personnel to diagnose current PTSD.

#### Other measures

The Social Constraints Scale (SCS) (Lepore & Ituarte, 1999) measures perceptions of negative social responses to trauma-related disclosure in the last week. The SCS has 15 self-reported items and yields a score ranging from 15 (no negative social support) to 60 (a lot of negative social support). As several participants (30%) mentioned no longer receiving any negative social support at the post-treatment, these data were dichotomized using a cut-off score of 16. The 54-item Social Adjustment Scale by Self-Report (SAS-SR) (Weissman & Bothwell, 1976) was used to measure social functioning. Finally, DSM-IV-TR axis I psychiatric comorbidity was assessed by a trained clinical psychologist with the semi-structured Mini International Neuropsychiatric Interview (MINI) (Lecrubier et al., 1997). A Glasgow Coma Scale (Teasdale & Jennett, 1974) score <13 upon arrival at the emergency room was used to operationalize the construct of “suspected or confirmed traumatic brain injury.” The on-line Injury Severity Score (http://www.trauma.org/archive/scores/iss.html) was used to obtain an individual's score reflecting the severity of the injuries sustained during the event. Substance use was assessed using the MINI supplemented by a short homemade questionnaire. Help-seeking behavior was evaluated by asking participants if they had requested the help of a series of mental health professionals and para-professionals since the event, and to what extent.

### The intervention

Developed and manualized by Cordova and Ruzek (2000), the intervention is fully described elsewhere (Cordova et al., 2003). This two-session dyadic intervention includes elements of psychoeducation and motivational interviewing, and targets communication between the patient and significant other, aiming to facilitate support, promote bi-directional disclosure, reduce disclosure-constraining behaviors, and improve coping. It promotes the disclosure of thoughts and emotions about the trauma in the natural environment of the dyad while attempting to reduce social constraints on disclosure and negative social support interactions. Examples of negative social support include comments directed toward the trauma survivors that may invalidate their experience or discourage future support seeking such as “Get over it” or “It wasn't that bad.”

The first intervention session has four objectives: (1) improve the knowledge of the process of adjustment to traumatic life experiences; (2) increase tolerance to the dysphoric emotions that are part of this process; (3) increase the amount of talking and sharing about the traumatic event, and instill a sense of motivation and self-efficacy for doing so; and (4) decrease social constraint behaviors in response the disclosure of thoughts and feelings about the traumatic event. During the therapy session, there was no requirement for re-telling the traumatic event in detail, or for experiencing strong trauma-related emotions.

The second session, with the trauma survivor and the significant other, was designed to increase the likelihood that the trauma survivor and dyadic partner would implement the objectives outlined in session one. Thus, the format is essentially a review of session one with an emphasis on the stumbling blocks encountered so far. The 10-step intervention (see Table 2) was designed to be offered at least 2 weeks after trauma exposure. Led by a social worker or a nurse trained and supervised every second week by a clinical psychologist, the initial 90-min session took place on average 26 days (SD *=*8.27) after the traumatic event. A 75-min second session was scheduled 2 weeks after the initial session. All the clinicians (6) received one full-day training. They were provided with a treatment manual that included a step-by-step synopsis on how to conduct the interview and on all the elements to cover. Sessions were audiotaped to monitor therapist treatment adherence to the treatment manual during the supervision sessions. Corrective measures, when needed, were implemented as an ongoing process. Adherence was deemed good to excellent for all therapists.

**Table 2:** Brief and early dyadic intervention summary

Protocol component	Description
Orientation	Purpose of the meeting: to share some of their reactions to the traumatic event and to anticipate potential problems and solutions during recovery.
Common reactions	Description and normalization of a wide variety of emotional or physical responses to trauma.
Importance of disclosing	Introduction and reinforcement of the idea that disclosing traumatic event-related thoughts and feelings to the dyadic therapy partner is a pivotal aspect of recovery.
Understanding avoidance	Discussion about the normal tendency to avoid distressing feelings and the negative impact of inhibiting disclosure.
Do's and don'ts	Ways of initiating disclosure, providing support, and responding to social constraints are discussed.
Motivational interview	Pros/cons of discussing traumatic event-related thoughts are examined as an attempt to instill motivation for disclosure and processing.
Discussion	Feedback elicited from the trauma survivor and the dyadic partner about what they have heard, disagreements and concerns they might have.
Referral information	Identify times when professional help should be sought.
	Referral information is given.
Summary	Review of the major points covered during the intervention.
Written material	Handouts reviewing the take-home messages of the meeting are provided.

### Procedure

The McGill Research and Ethics Board approved the study protocol. Prospective participants were identified by a member of the clinical staff on the basis of trauma exposure and asked if they would mind talking with a member of a research team. Participants meeting the study inclusion criteria were consented around the 10th day after trauma exposure and given the questionnaires with a stamped return envelope. Those returning their package were blindly randomized to either one of the two study conditions with a 50% chance. The intervention was provided at the hospital. Those in the no-intervention group simply filled out and returned the questionnaires. Participants completed the questionnaires approximately 21 days post-trauma (pre-treatment assessment), and 35 days post-trauma (between the first and second sessions of the intervention) and 3 months post-trauma (post-treatment assessment). Three months after trauma exposure, a diagnostic interview was scheduled at the research center or at the participant's home. The assessments were audiotaped in order to conduct interrater reliability checks; 20% of all the tapes were blindly rated by a trained research assistant for diagnostic accuracy (PTSD positive or negative). A 100% percent diagnostic agreement was obtained. Participants received 55 CAD$ for their time and effort. Although they could receive it at the end of the study protocol, only one participant from the control group requested the intervention.

#### Statistical methodology

An *a priori* power analysis based on pilot data was conducted using the PS software version 2.1.30 (Dupont & Plummer, 1997). With an equal sample size of 35 per cell, we had an 80% chance of detecting an effect size of *d=*0.67, in a two-sided *t*-test using an alpha level of 0.05 with self-reported PTSD symptoms as the outcome measure at the post-treatment.

To assess the primary hypothesis, a mixed approach, the growth curves model, was used, allowing measuring change over time in the intensity of the (IES-R) symptoms, at both the sample and individual levels. This approach has the advantage that data are truly modeled at the individual level. As IES-R score was measured at three time points (pre-treatment, mid-treatment, and post-treatment), patients with at least two time points were retained in the ITT analysis to support the linear assessment at the individual level. An additional grouping term was added to the model to evaluate if the trajectories of symptom's intensity differed depending on whether or not the patients attended the intervention sessions (time*group). Different types of matrix structure were tested to obtain the best fit to the variance–covariance matrix structure. To complement results interpretation, mean IES-R scores were compared between the groups (treated vs. untreated) at each time point, based on contrasts analysis applied on the interaction term time*group within the mixed-model repeated-measure ANOVA performed on the square-root of IES-R measurement. Descriptive within-group effect sizes were also derived.

To assess the secondary hypotheses, the number of participants (%) with a PTSD diagnosis at the post-treatment was compared between the groups using a Chi-square test. Cochran's *Q* test was also used to evaluate the pre- and post-decrease in the number of treated patients receiving negative social support. The same test was performed in the control group. Moreover, a Spearman correlation was calculated at post-treatment between the social support and the severity of the PTSD symptoms.

Secondary analyses also included the type of dyadic partners, the occupational functioning, and substance use as well as the comorbidity and other concurrent interventions. Two-sided Student's *t*-tests were performed to compare the CAPS and IES-R measures between treated patients accompanied by their partner and those accompanied by someone else. The proportion of treated patients working during the intervention session was compared to the same proportion in the untreated group using Fisher's exact test. A mixed-model repeated-measure ANOVA was also performed to compare post-treatment to pre-treatment substance use. The model included time, group and the interaction time*group. All statistical tests were performed at an alpha level of 5% in a two-sided test.

## Results

### ITT population

#### Results of the growth curves analysis

There was a statistically significant time-by-group interaction, *t*(71)=2.81, *p*=0.006. More specifically, the estimated coefficient was −0.51 and represented the estimated differential in the rate of change between both groups for the square root of IES-R score (treated rate −0.75 vs. untreated rate −0.24). The treatment group coefficient was 0.47 (*p*=0.34). This is the estimated differential in the initial square root of IES-R score between treated and untreated patients.

#### Results of the mixed-model repeated-measure ANOVA

**Fig. 2:**
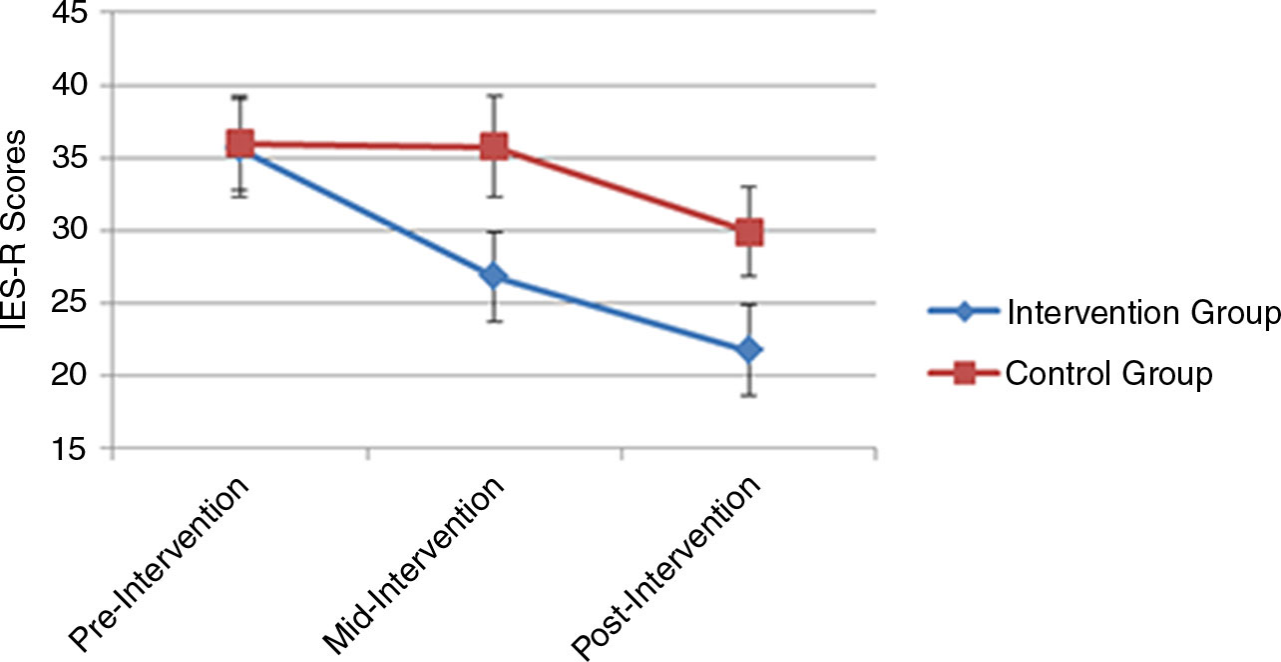
Self-reported mean PTSD symptom scores across time for the two study groups according the IES-R.

There was a statistically significant time-by-group interaction, *F*(2,72)=4.17, *p*=0.019. The contrasts analysis on the interaction term demonstrates that the PTSD symptom difference, as measured by the square-rooted IES-R, between the groups tend to increase with time (see Fig. 2 and Table 3) and achieved significance after the intervention treatment was completed, *F*(1,72)=4.44, *p*=0.039.

**Table 3:** Means and standard deviation of IES-R for every measurement time: intent-to-treat and per-protocol samples

Group	Pre-intervention *M* (SD)	*p* 1	Mid-intervention *M* (SD)	*p* 1	Post-intervention *M* (SD)	*p* 1
Intent-to-treat						
Untreated (*n*=37)	36.01 (19.90)	0.98	35.77 (21.27)	0.15	29.90 (18.38)	0.04
Treated (*n*=37)	35.64 (20.25)		26.82 (18.33)		21.73 (18.90)	
Per-protocol						
Untreated (*n*=32)	35.48 (18.64)	0.94	34.95 (20.47)	0.18	30.32 (17.95)	0.05
Treated (*n*=34)	35.25 (19.07)		25.99 (16.29)		22.74 (18.94)	

Pre-intervention assessment was conducted 10 days post-trauma; the first intervention was conducted 21 days post-trauma; the mid-intervention assessment was conducted immediately before the second session, 35 days post-trauma; the post-intervention assessment was conducted 3 months post-trauma.

1 *p*-Value resulting from contrasts analysis on the interaction term time*group within the mixed-model ANOVA (repeated-measure) performed on the square root of the IES-R measurement.

As hypothesized, the social support variable at the post-treatment was well correlated with the severity of PTSD symptoms at the post-treatment, Spearman correlation of 0.45 (*p*<0.001).

### Per-protocol population

#### Results of the growth curves analysis

There was a statistically significant time-by-group interaction, *t*(65)= 2.53, *p*=0.014. More specifically, the estimated coefficient was −0.50 and represented the estimated differential in the rate of change between both groups for the square root of IES-R score (treated rate −0.72 vs. untreated rate −0.22). The treatment group coefficient was 0.48 (*p*=0.351).

#### Results of the mixed-model repeated-measure ANOVA

There was a statistically significant time-by-group interaction, *F*(2,64)=3.30, *p*=0.043.

In the ITT sample, using Table 3 data, we calculated a within-group effect size for each group using Cohen's *d* method (Cohen, 1988) and observed a *d=*0.71 for the intervention and a *d=*0.32 for the control group. By subtracting the effect size observed in the control group from the one observed in the intervention group, a medium effect size of *d=*0.39 was obtained. Those figures for the per-protocol population were *d*=0.66, 0.28, and 0.38.

### Secondary analyses

Secondary analyses were conducted on the per-protocol sample. In spite of a significant between-group symptom reduction, a Chi-square analysis revealed no significant difference in the number of participants with a CAPS PTSD diagnosis at the post-treatment in the control group (8/32) compared to that of the intervention group (5/34), χ^2^(1, *N=*66)=1.10, *p=*0.36. These results did not change when the IES-R clinical cut-off score of 33 for PTSD (Creamer, Bell, & Failla, 2003) was used instead.

We observed a significant pre- and post-decrease (Cochran's *Q* test) in the number of participants who received negative social support in the intervention group, χ^2^(2, 10.40), *p=*0.006. This was not found in the control group, χ^2^(2, 1.20), *p=*0.55. At the post-treatment, seven participants in the control group reported no longer receiving any negative social support, while this was the case for 13 participants in the intervention group. The social support variable at the post-treatment group was well correlated (*r=*0.44, *p<*0.001) with the severity of PTSD symptoms at the post-treatment.

#### Dyadic partners: who were they?

All of the participants currently involved in a relationship (23 out of the 34 participants) attended the intervention with their life partner. Eight single participants came with a family member and three came with a friend. A *t*-test failed to find any significant difference in the two PTSD symptom measures, namely the CAPS, *t*(32)=1.32, *p=*0.20 and the IES-R, *t*(32)=1.77, *p=*0.09 between those who came with their life partner and those who were accompanied by another type of partner.

#### Occupational functioning and substance use

Thirty-eight participants had a job before the traumatic event. Fisher's exact test on the SAS-SR scores demonstrated that significantly more participants in the intervention than in the control group continued to work between the pre-treatment and the post-treatment (9 vs. 1, *p=*0.01). A repeated-measure ANOVA with the substances use questionnaire scores at pre-treatment and post-treatment showed no time effect *F*(1,64)=2.27, *p=*0.14, no group effect *F*(1,64)=0.82, *p=*0.37 and no time-by-group interaction, *F*(1,64)=3.70, *p=*0.06, although a trend was noted.

#### Comorbidity and other concurrent interventions

The development of axis I comorbidity was assessed during the study. No statistical tests were conducted on this data as a means to reduce the multiplicity of tests. The figures are very low in both groups. In the intervention group, one participant suffered from major depression versus two in the control group. Two participants in the intervention versus one participant in the control group suffered from panic disorder without agoraphobia. One participant in the intervention group and two participants in the control group suffered from panic disorder with agoraphobia. Two participants from the control group developed social phobia. Only one participant in the control group had obsessive–compulsive disorder and one other participant in the intervention group had a disorder of generalized anxiety. During the study, 10 (29%) participants in the intervention group and nine (28%) participants in the control group sought help from mental health (para-)professionals, χ^2^(1); 0.91, *p=*0.56.

## Discussion

The primary objective of this study was to empirically test the efficacy of a brief dyadic secondary prevention intervention, as described by Cordova et al. (2003), in reducing PTSD symptomatology in the weeks and months following trauma exposure. Individuals presenting to the emergency room after trauma exposure who received the intervention were less symptomatic 3 months later compared to those who did not receive it. In part because of the small sample size and limited power, this difference did not translate into a significant difference at the diagnostic level in spite of the PTSD rate being cut by almost 50% (25% vs. 14% PTSD). The number of individuals perceiving negative social support from their significant others at the post-treatment was also lower among those who received the intervention, and this reduction was not observed in the control group.

Those encouraging results are in sharp contrast with the results of several previous randomized controlled trials (National Institute for Clinical Excellence, 2005) that failed to find significant benefits in participating in an early single-session individual post-traumatic prophylactic intervention over the natural rate of recovery. The study's effect size of *d=*0.39, adjusted for the effect of time, is consistent with that observed for other non-CISD (Critical Incident Stress Debriefing) effective interventions, or with CISD group interventions (Van Emmerik, Kamphuis, Hulsbosch, & Emmelkamp, 2002).

Our study is unique in that participants were recruited on the basis of exposure criteria A1 and A2 for PTSD: we included only individuals who experienced a life-threat *and* who reported a significant peritraumatic distress reaction. We did not include individuals for whom the event turned out to be only a stressful (but non-traumatic) “critical incident.” This is also reflected in the relatively high rates of PTSD observed 3 months after trauma exposure. This methodological choice ensured that only those deemed more likely to benefit from an intervention were included in the study. This also reduced the “noise” associated with spontaneous recovery among the groups compared to other studies and improved our power to reject the null hypothesis. We believe that several studies examining the efficacy of early interventions were insufficiently powered because of the issue of spontaneous recovery. However, even in such a sample (made of individuals meeting the A1 and A2 criteria) it is expected that about 90% of individuals will not develop PTSD (e.g., Breslau et al., 1998).

For ethical reasons, we could not limit use of other mental health resources or psychotropic medications. However, between pre- and post-treatment, use of other sources of help was equivalent in the two groups.

Contrary to some other types of early intervention, this one was provided on the third or fourth week after trauma exposure. We believe that in the first days following exposure, individuals may be relatively non-receptive toward receiving an intervention to prevent the development of PTSD (but see Rothbaum et al., 2012), for a variety of reasons including the primacy of other pressing health, legal or family concerns. After a few weeks, individuals may be more willing to accept an appointment to see someone once they begin to realize that they are experiencing disturbing PTSD symptoms that are not going away.

Ideally, early interventions should be effective in reducing the development of mental health problems and problems in functioning; be relatively brief to deliver, capable of application by a wide range of potential helpers, and easy to teach and disseminate. The current intervention was composed of two contacts lasting less than 2 h each. A second session was included due to an established lack of evidence for single-session interventions (Bisson, 2003) with the intent of increasing the commitment to implement the elements discussed in the first session. We believe we achieved this by giving the dyadic partners 1–2 weeks to try out a number of coping strategies and conveying the expectation that they would come back to check in and report on this “experiment,” and make adjustments based on their experience.

Importantly, the intervention was delivered by nurses and social workers of the hospital where the patients had been admitted, so that this study speaks to the effectiveness of the intervention rather than its efficacy because it is provided in a natural setting. As many patients had medical follow-up appointments at the hospital, from a practical point of view it made sense to plan the intervention at the same institution. We believe an initial rapport had been developed, if not with the nurse or social worker, at least with the institution itself. Furthermore, the relative simplicity of the intervention enabled rapid training (1 day of training time) and implementation was adherent to the intervention protocol.

To date, early interventions have focused largely on the individual trauma survivor. The present study departs from this tradition to focus on the survivor and a significant other. As the environments in which recovery must occur are social in nature (Lepore, Silver, Wortman, & Wayment, 1996), it may be useful to explore interventions that focus on social interactions, especially since traumas also affect the well-being of those close to the survivor.

Participants from the intervention group reported a decrease in the amount of negative social support coming from their dyadic partner at the post-treatment. These results are consistent with findings that emphasize the importance of social support as a predictor of unremitting PTSD symptoms (Guay, Billette, & Marchand, 2006; Ozer, Best, Lipsey, & Weiss, 2003). However, this study was not designed to test the mechanisms underlying the efficacy of the intervention (see e.g. Olff, Langeland, Witteveen, & Denys, 2010). More work will be needed to investigate whether the intervention effectively addressed the frequently observed relationship between social support and PTSD.

Finally, it is significant that the intervention affected workplace functioning outcomes. Effects on role functioning and quality of life should receive more attention in the future.

In summary, from a clinical perspective, we showed that a brief, early, and effective intervention *can* be provided by nurses or social workers in hospital settings, at a fairly low cost to individuals presenting to the emergency room as the result of trauma exposure. Although the study involved mostly survivors of a motor vehicle accident, the intervention might be useful following exposure to a range of traumatic events. Furthermore, the fact that slightly more women (*p=*0.09) ended up in the intervention group probably worked against confirmation of our main hypothesis since women are known to have a PTSD rate twice as high as that of men and to recover more slowly (Breslau et al., 1998).

### Limitations and future direction

Multiple tests were conducted without correcting for the inflation in alpha error. Caution is therefore required since results may be less stable.

Although individuals receiving the treatment expect to improve while those in the no-intervention group do not, many early intervention studies that have used waitlist controls have failed to produce significant intervention effects. Therefore, this expectation is not sufficient enough to explain the observed results. Nonetheless, it remains plausible that the experimental group improved as a result of the interventions it received outside of the protocol. However, this is unlikely since the same number of individuals in both groups received some treatments outside of the protocol.

Participants were relatively uniform in terms of culture/ethnicity and socio-economic status. Generalization to other groups cannot be assumed.

No data were collected about the number of participants who were approached at the emergency room to participate in the study. This could alter the external validity of the study. However, as a first efficacy intervention, we opted to emphasize internal validity.

Given that treatment efficacy decreases with the passage of time for cognitive-behavioral therapy for PTSD (Bradley, Greene, Russ, Dutra, & Westen, 2005), a follow-up longer than 3 months is needed to demonstrate the usefulness of this intervention. Furthermore, the deleterious effects of debriefing have been found more than 12 months post-trauma (Bisson, Jenkins, Alexander, & Bannister, 1997; Mayou, Ehlers, & Hobbs, 2000). It will be interesting to monitor the long-term effects of this intervention.
